# A prospective cohort study of health behavior profiles after age 50 and mortality risk

**DOI:** 10.1186/1471-2458-12-803

**Published:** 2012-09-18

**Authors:** Benjamin A Shaw, Neda Agahi

**Affiliations:** 1 School of Public Health, University at Albany, One University Place, Rensselaer, NY, 12144, USA; 2 Aging Research Center, Karolinska Institutet/Stockholm University, Gavlegatan 16, 113 30, Stockholm, Sweden

## Abstract

**Background:**

This study examines the mortality risk associated with distinct combinations of multiple risk behaviors in middle-aged and older adults, and assesses whether the mortality risks of certain health behaviors are moderated by the presence of other risk behaviors.

**Methods:**

Data for this prospective cohort study are from the Health and Retirement Study (HRS), a nationwide sample of adults older than 50 years. Baseline data are from respondents (n = 19,662) to the 1998 wave of the HRS. Twelve distinct health behavior profiles were created, based on each respondent’s smoking, physical activity, and alcohol use status in 1998. Mortality risk was estimated through 2008 using Cox regression.

**Results:**

Smoking was associated with elevated risk for mortality within all behavioral profiles, but risk was greatest when combined with heavy drinking, both for middle-aged (ages 51–65) and older (ages 66+) adults. Profiles that included physical inactivity were also associated with increased mortality risk in both age groups. However, the impact of inactivity was clearly evident only among non-smokers; among smokers, the risk of inactivity was less evident, and seemingly overshadowed by the risk of smoking. Moderate drinking was protective relative to abstinence among non-smokers, and relative to heavy drinking among smokers.

**Conclusions:**

In both middle-aged and older adults, multiple unhealthy behaviors increase mortality risk. However, the level of risk varies across unique combinations of unhealthy behaviors. These findings highlight the role that lifestyle improvements could play in promoting healthy aging, and provide insight into which behavioral combinations should receive top priority for intervention.

## Background

Over the past two decades, research evidence has persistently shown that roughly one-half of annual deaths in the United States are attributable to personal behaviors [1]. More recently, research has shown that the health hazards of major risk behaviors, such as smoking, heavy alcohol consumption, and physical inactivity, are evident not just among young and middle-aged adults, but also among older adults [2-5]. Strong linkages have also been reported between positive health habits and the postponement of mobility impairment and disability in later life [6-9].

An increasingly common approach to studying health behavior risks involves a focus on the combined effects of multiple unhealthy behaviors. Most commonly, researchers have created a combined health behavior index, where dichotomous measures of multiple behaviors are summed e.g., [4,6,10-23]. Others have attempted to account for the risks associated with various individual behaviors more precisely by defining multiple categories of risk for each behavior before creating a composite measure [24,25], or by assigning differential weights for each behavior prior to creating a summary score [26]. Findings from these types of studies generally show that mortality can be postponed by maximizing the number of healthy behaviors in one’s profile, and minimizing the number of unhealthy, or risky, behaviors.

Notwithstanding the value of these studies, they have not been able to differentiate between the portions of risk attributable to each of the individual health behaviors, assess the potential for healthy behaviors to moderate the impact of other risky behaviors, or identify particularly dangerous combinations of risk behaviors. In this study we address these issues by examining the mortality risk associated with several person-centered configurations of multiple behaviors [27], or “health behavior profiles”, each representing a different combination of status with respect to three key behaviors: smoking, alcohol use, and physical inactivity. These particular behaviors are among the most widely recognized behavioral risks in the adult population [1,28,29], but their joint effects on mortality have not been sufficiently examined. Moreover, while some studies have found these behaviors to be correlated with one another [30], others have found that these health behaviors tend to be only weakly correlated [31,32], and thus do not present as a unidimensional construct. This suggests the potential value of moving beyond the examination of cumulative indices of health risk behaviors by adopting a person-centered approach that allows for consideration of how unique combinations of multiple health behaviors may be differentially associated with survival in later life [29,31,33-36].

We hypothesize that while each risk behavior will present some degree of risk, and while the presence of multiple harmful behaviors will be associated with increasingly elevated mortality risk, not all multiple combinations of unhealthy behaviors are likely to be associated with the same level of mortality risk. We suspect that differences in survival observed across unique configurations of multiple unhealthy behaviors are a reflection of variations in the joint effects, or interactions, between different combinations of health behaviors. Consistent with theories of disadvantage and vulnerability [37], some combinations of multiple risk behaviors could result in compounding effects, whereby the risks of one unhealthy behavior are magnified in the presence of another unhealthy behavior. Alternatively, using reasoning similar to that espoused by the “Blaxter hypothesis” – which posits that socially disadvantaged groups may be less vulnerable than advantaged groups to the risks of unhealthy behaviors [38-40] – one might expect that the effects of some unhealthy behaviors may be attenuated, or overshadowed, in the presence of the damage caused by other unhealthy behaviors.

Prior research has described the clustering of various behaviors into discrete health lifestyles [19,29,31,33-35,41-44], and some have examined the impact of combinations of multiple behaviors on specific health conditions [20,45]. Studies focusing on the mortality risk associated with unique combinations of multiple behaviors have been conducted within special populations, such as elderly male physicians [36], or middle-aged Scottish men [46]. More recently, using data from the National Health and Nutrition Examination Survey (NHANES), researchers have estimated the mortality risk of various combinations of health behaviors among representative samples of adults aged 17 years and older [10], and ages 20 and older [11]. However, the current study is unique in that it examines the potential joint effects of three important health risk behaviors using a population-based, nationally representative sample of adults over the age of 50. By focusing on the mortality risk of multiple behavior profiles within both middle-aged and older adults, the current study can help to determine whether the mortality risk of various high risk behavioral profiles is sustained in later life, or if this risk attenuates among those who survive into old age, perhaps due to selective survival.

## Methods

### Data source

Data for this study came from the Health and Retirement Study (HRS), a nationally representative panel survey of community-dwelling older Americans. The HRS data and supporting documentation are publicly accessible [47]. The original HRS sample, born between 1931–41, was selected in 1992 from a sampling frame of households, generated using a multi-stage clustered area probability frame. In 1993, an additional cohort (the Asset and Health Dynamics among the Oldest Old, or AHEAD, cohort), born between 1890–1923, was sampled and interviewed. Then, in 1998, these two samples were merged. A total of 10,584 respondents from original HRS cohort were interviewed in 1998, for a response rate 86.7%. The sample size for the AHEAD cohort in 1998 was 5,591, and the response rate was 91.4%. Two additional cohorts were also included in 1998: the War Babies (WB) cohort included 2,529 individuals born between 1942 and 1947, and the response rate in 1998 was 69.9%. The Children of the Depression Age (CODA) sample included 2,320 individuals born between 1924 and 1930, and the response rate for this sample in 1998 was 72.5%.

Baseline data for the current study come from 1998. Respondents from the HRS cohort, and AHEAD respondents younger than 80, were surveyed over the phone, although face-to-face interviews were used when there was no telephone in the household or when the respondent’s health limitations prevented them from completing an hour-plus telephone session. The baseline interviews for CODA and WB cohorts, as well as AHEAD cohort respondents 80 years old or older, were conducted face-to-face. A total of 21,384 respondents were interviewed in 1998. Respondents included both age-eligible persons from selected households, plus their partners or spouse, regardless of age-eligibility. For the current analyses, this sample was restricted to those 51 years old or older in 1998 (N = 20,249). After excluding individuals with incomplete data regarding health behaviors (1.6%), the total sample included 19,914 respondents. Mortality status was assessed through the end of 2008. During this 10-year period, a total of 5,857 individuals (29.4%) died. As described below, 252 participants who died during the baseline year were excluded from the analyses; as a result, the analytic sample includes 19,662 participants, 5,605 (28.5%) of whom died before the end of 2008.

All analyses utilize the HRS respondent-level population weights. This weight variable is scaled so as to yield weight sums which correspond to the number of individuals in the U.S. population as measured by the Current Population Survey from March 1998. We normalized the weight variable by dividing it by its mean in order to scale it to the current study’s sample size.

### Measures

#### Health behavior profiles

For each respondent, a multi-behavior profile was created based on self-reported smoking, physical activity, and alcohol consumption at baseline (1998). Smoking was measured dichotomously (current smoker or not); at baseline, 17.2% of respondents identified themselves as current smokers.

Physical activity was measured with the following survey item: “On average over the last 12 months have you participated in vigorous physical activity or exercise three times a week or more? By vigorous physical activity, we mean things like sports, heavy housework, or a job that involves physical labor.” Responses were coded in a binary format (0 = No; 1 = Yes); at baseline, 55.8% of respondents answered “No” and were classified as inactive.

Alcohol consumption was coded as abstinence, moderate drinking, and heavy drinking, based on the self-reported number of alcoholic drinks a respondent consumed per week. We followed guidelines from the National Institute of Alcohol Abuse and Alcoholism [48] for older adults and defined heavy drinking as consuming an average of more than 7 drinks per week. At baseline, 9.5% of respondents were defined as heavy drinkers. Respondents who drank less were defined as moderate drinkers (22.1%), and respondents who reported no drinking were defined as non-drinkers (68.4%). Moderate alcohol consumption was used as the reference group.

By identifying all possible combinations of these 3 health behavior measures, 12 unique health behavior profiles were defined, and subgroups of respondents were formed according to their shared multi-behavioral profiles.

#### Mortality

Follow-up on mortality status was carried out from baseline through the end of the year 2008. Date of death (month and year) was obtained from the National Death Index.

#### Control variables

Baseline age, gender, race, education, household income, and marital status were included in the analyses. Also, the effects of baseline health were controlled for with measures of self-rated health, self-reported presence of 5 serious chronic conditions (i.e., high blood pressure, diabetes, cancer, heart disease, or stroke), a count of functional limitations, and weight status (with underweight defined as body mass index (BMI) < 18.5 and overweight as BMI ≥ 25).

### Data analysis

The analyses compared the risks of dying over a 10-year period for respondents with different health behavior profiles, using Cox regression. First, the mortality risk of each health behavior was assessed with a model that included all of the individual behaviors specified as separate variables. Then, the mortality risks of each health behavior profile, relative to a risk-free profile, were estimated. These models were estimated for the sample as a whole, and also after stratifying the sample into two age groups: ages 51–65 and 66+ at baseline. Next, in order to compare the risk of mortality between specific behavioral profiles, a series of hazard models was estimated using different reference groups.

All models were adjusted for differences in sociodemographic characteristics and baseline health problems. As a further control for the effects of baseline health status on health behaviors, all respondents who died during the baseline year (1998) were excluded from the analyses. Because preliminary analyses showed minimal gender differences in the mortality risks associated with the 12 health behavior profiles, all analyses were conducted with data from men and women pooled.

## Results

Descriptive characteristics of each of the 12 health behavior profiles are presented in Table 1. The most prevalent profile was characterized by non-smoking, inactivity, and abstention from alcohol (34.1%). The least prevalent profile was characterized by smoking, vigorous activity, and heavy drinking (1.4%). Furthermore, sociodemographic characteristics, as well as baseline self-rated health and BMI, varied considerably across the profiles (p < .001 for all omnibus tests of sociodemographic and health differences across profiles). For example, the non-drinking profiles tended to be dominated by women (ranging between 54.4-66.8%), whereas the heavy drinking profiles tended to be dominated by men (ranging between 66.9-76.6%). Age differences between profiles were also evident, with average baseline ages ranging from 58.8 years in the smoking, active, heavy drinking profile, to 68.5 years in the non-smoking, inactive, non-drinking profile. Poor self-rated health was more prevalent in the smoking profiles; however, closer examination reveals considerable variation across both the non-smoking and smoking profiles. Poor self-rated health was particularly common in the inactive, non-drinking profiles for both smokers (48.4%) and non-smokers (41.8%), and relatively uncommon among the physically active, alcohol consuming profiles (13.9-15.7% for smokers; 9.6%-11.0% for non-smokers).

**Table 1:** Characteristics of the 12 health behavior profiles (U.S. adults over age 50 in 1998)

	**Non-smokers**						**Smokers**					
	**Active, mod. drinker**	**Active, non-drinker**	**Active, heavy drinker**	**Inactive, mod. drinker**	**Inactive, non-drinker**	**Inactive, heavy drinker**	**Active, mod. drinker**	**Active, non-drinker**	**Active, heavy drinker**	**Inactive, mod. drinker**	**Inactive, non-drinker**	**Inactive, heavy drinker**
**N**	**1,986**	**4,662**	**664**	**1,684**	**6,702**	**594**	**286**	**820**	**274**	**384**	**1,274**	**332**
%	10.1	23.7	3.4	8.6	34.1	3.0	1.5	4.2	1.4	2.0	6.5	1.7
Gender^a^												
% Women	45.1	57.1	25.0	51.1	66.8	23.4	44.1	54.4	27.4	54.4	62.9	33.1
% Men	54.9	42.9	75.0	48.9	33.2	76.6	55.9	45.6	72.6	45.6	37.1	66.9
Mean Age^a^ (SD)	63.5 (9.2)	65.1 (9.7)	63.1 (8.4)	66.4 (11.1)	68.5 (11.2)	64.8 (9.6)	59.2 (7.0)	59.8 (7.6)	58.8 (7.0)	61.0 (8.5)	62.6 (8.9)	61.3 (8.0)
Mean Education^a^ (SD)	13.7 (2.7)	12.2 (3.1)	13.7 (2.7)	13.2 (2.9)	11.5 (3.5)	13.0 (3.0)	12.5 (2.7)	11.7 (2.8)	12.5 (3.0)	12.5 (3.0)	11.3 (3.2)	12.1 (2.9)
Mean Household Income, x 1000^a^ (SD)	83.9 (137.9)	50.4 (61.8)	83.7 (111.3)	70.7 (153.3)	38.0 (49.6)	100.1 (462.7)	60.1 (58.0)	44.3 (53.6)	68.6 (119.8)	56.8 (93.7)	32.3 (37.7)	59.2 (93.3)
Marital status^a^												
% Married	78.3	69.3	82.7	72.3	59.1	77.2	66.1	61.6	63.9	59.8	52.6	65.7
% Unmarried	21.7	30.7	17.3	27.7	40.9	22.8	33.9	38.4	36.1	40.2	47.4	34.3
Race^a^												
% White	94.4	89.1	94.4	94.1	85.5	93.6	87.8	84.9	89.8	88.0	82.7	90.1
% Other	5.6	10.9	5.6	5.9	14.5	6.4	12.2	15.1	10.2	12.0	17.3	9.9
Self-rated health^a^												
% G/VG/Ex	90.4	79.7	89.0	77.3	58.2	75.9	84.3	76.3	86.1	68.8	51.6	72.3
% Fair/Poor	9.6	20.3	11.0	22.7	41.8	24.1	15.7	23.7	13.9	31.3	48.4	27.7
Body Mass Index^a^												
% Underweight	1.2	1.2	0.3	1.3	2.5	0.7	1.0	2.1	1.5	5.0	5.9	3.3
% Normal	41.5	36.5	35.7	34.5	31.3	30.5	49.7	44.4	50.7	42.7	39.7	47.9
% Overweight	57.3	62.3	64.0	64.2	66.1	68.8	49.3	53.6	47.8	52.4	54.4	48.8

^a^ p ≤ .001 based on chi-square tests for differences in gender, marital status, race, self-rated health, and body mass index across health behavior profiles, and F-tests (ANOVA) for differences in age, education, and household income.

Tables 2 and 3 present results from the hazard models. In the top portion of Table 2, associations between each health behavior and mortality are presented in order to show the average, independent, effects of these behaviors. Results from analyses with the full sample indicate that each health risk behavior was independently associated with an elevated risk for mortality. The mortality risk associated with smoking was the highest among the behaviors (hazard ratio (HR) = 1.9, 99% confidence interval (CI) = 1.7-2.1), suggesting that independent of the other health behaviors, the risk of dying among smokers was nearly twice as high as the risk for non-smokers. The risk of dying was about 40% higher among inactive compared to active respondents (HR = 1.4, 99% CI = 1.3-1.5). Compared to moderate drinkers, the risk of dying was about 30% higher, both for non-drinkers (HR = 1.3, 99% CI = 1.2-1.4) as well as heavy drinkers (HR = 1.3, 99% CI = 1.1-1.5). This pattern of findings was similar for both middle-aged and older adults.

**Table 2:** **Mortality risk (1999–2008) associated with health behaviors and health behavior combinations measured in 1998**^a^

	**Full sample**			**Ages 51--65**			**Ages 66+**		
	**HR (99% CI)**								
**Individual behaviors**									
Current smoker	1.9	***	(1.7, 2.1)	2.1	***	(1.8, 2.5)	1.8	***	(1.6, 2.0)
Inactive	1.4	***	(1.3, 1.5)	1.4	***	(1.2, 1.7)	1.4	***	(1.3, 1.5)
Alcohol use									
-Moderate	1.0	(Ref)		1.0	(Ref)		1.0	(Ref)	
-Abstain	1.3	***	(1.2, 1.4)	1.3	***	(1.0, 1.6)	1.3	***	(1.1, 1.4)
-Heavy	1.3	***	(1.1, 1.5)	1.3	**	(1.0, 1.7)	1.2	**	(1.0, 1.5)
**Health behavior profiles**									
Non-smokers									
Active, moderate drinker	1.0	(Ref)		1.0	(Ref)		1.0	(Ref)	
Active, non-drinker	1.4	***	(1.1, 1.6)	1.4	*	(0.9, 2.1)	1.3	***	(1.1, 1.6)
Active, heavy drinker	1.0		(0.7, 1.3)	1.0		(0.5, 2.0)	1.0		(0.7, 1.4)
Inactive, moderate drinker	1.5	***	(1.2, 1.8)	1.8	***	(1.1, 2.9)	1.4	***	(1.1, 1.8)
Inactive, non-drinker	1.9	***	(1.6, 2.2)	2.3	***	(1.6, 3.4)	1.8	***	(1.5, 2.2)
Inactive, heavy drinker	1.7	***	(1.3, 2.2)	1.7	*	(1.0, 3.0)	1.7	***	(1.3, 2.3)
Smokers									
Active, moderate drinker	1.9	***	(1.2, 2.9)	2.7	***	(1.4, 5.1)	1.7	*	(0.9, 3.1)
Active, non-drinker	2.7	***	(2.1, 3.4)	3.4	***	(2.2, 5.4)	2.4	***	(1.7, 3.4)
Active, heavy drinker	3.4	***	(2.4, 4.8)	5.0	***	(3.0, 8.4)	2.6	***	(1.5, 4.6)
Inactive, moderate drinker	2.7	***	(2.0, 3.6)	3.5	***	(2.1, 5.9)	2.4	***	(1.6, 35)
Inactive, non-drinker	3.2	***	(2.6, 3.9)	3.9	***	(2.6, 5.9)	3.0	***	(2.3, 3.8)
Inactive, heavy drinker	4.2	***	(3.1, 5.6)	4.8	***	(3.0, 7.8)	4.1	***	(2.8, 6.1)

*P ≤ .05; ** *P* ≤ .01; *** *P* ≤ .001.

^a^Models adjusted for the following in 1998: age, gender, marital status, race, education, household income, self-rated health, 5 serious chronic diseases, functional limitations, and body mass index.

**Table 3:** **Mortality risk among adults over 50: the effects of health behaviors within different behavioral profiles**^a^

	**Mortality risk of:**			
	**Smoking**	**Physical inactivity**	**Non-drinking**	**Heavy drinking**
**Behavioral profiles**	**HR (99% CI)**			
Non-smokers				
Active, moderate drinker				
Active, non-drinker			1.4***	
			(1.1, 1.6)	
Active, heavy drinker				1.0
				(0.7, 1.3)
Inactive, moderate drinker		1.5***		
			(1.2, 1.8)	
Inactive, non-drinker			1.4***	1.3***	
		(1.3, 1.5)	(1.1, 1.4)		
Inactive, heavy drinker		1.8***		1.1	
		(1.2, 2.5)		(0.9, 1.4)	
Smokers					
Active, moderate drinker	1.9***				
	(1.2, 2.9)				
Active, non-drinker	2.0***		1.4*		
	(1.6, 2.4)		(0.9, 2.2)		
Active, heavy drinker	3.6***			1.8**	
	(2.4, 5.4)			(1.1, 2.9)	
Inactive, moderate drinker	1.8***	1.4			
	(1.3, 2.4)	(0.9, 2.3)			
Inactive, non-drinker	1.7***	1.2*	1.2		
	(1.5, 2.0)	(1.0, 1.5)	(0.9, 1.6)		
Inactive, heavy drinker	2.5***	1.2		1.6***	
	(1.8, 3.4)	(0.8, 1.68)		(1.1, 2.2)	

* *P* ≤ .05; ** *P* ≤ .01; *** *P* ≤ .001.

^a^Models adjusted for the following in 1998: age, gender, marital status, race, education, household income, self-rated health, 5 serious chronic diseases, functional limitations, and body mass index.

In the bottom portion of Table 2, associations between each health behavior profile and mortality are presented, with non-smoking, physically active, moderate drinkers serving as the reference group. In the full sample, as well as both age groups, all but one behavioral profile was associated with elevated mortality risk. Only among non-smoking, physically active, heavy drinkers was mortality risk not elevated. Among the other non-smoking profiles in the full sample, the risk of mortality was elevated by between 40%, for non-smoking, physically active, non-drinkers, and 90%, for non-smoking, inactive, non-drinkers.

**Figure 1:**
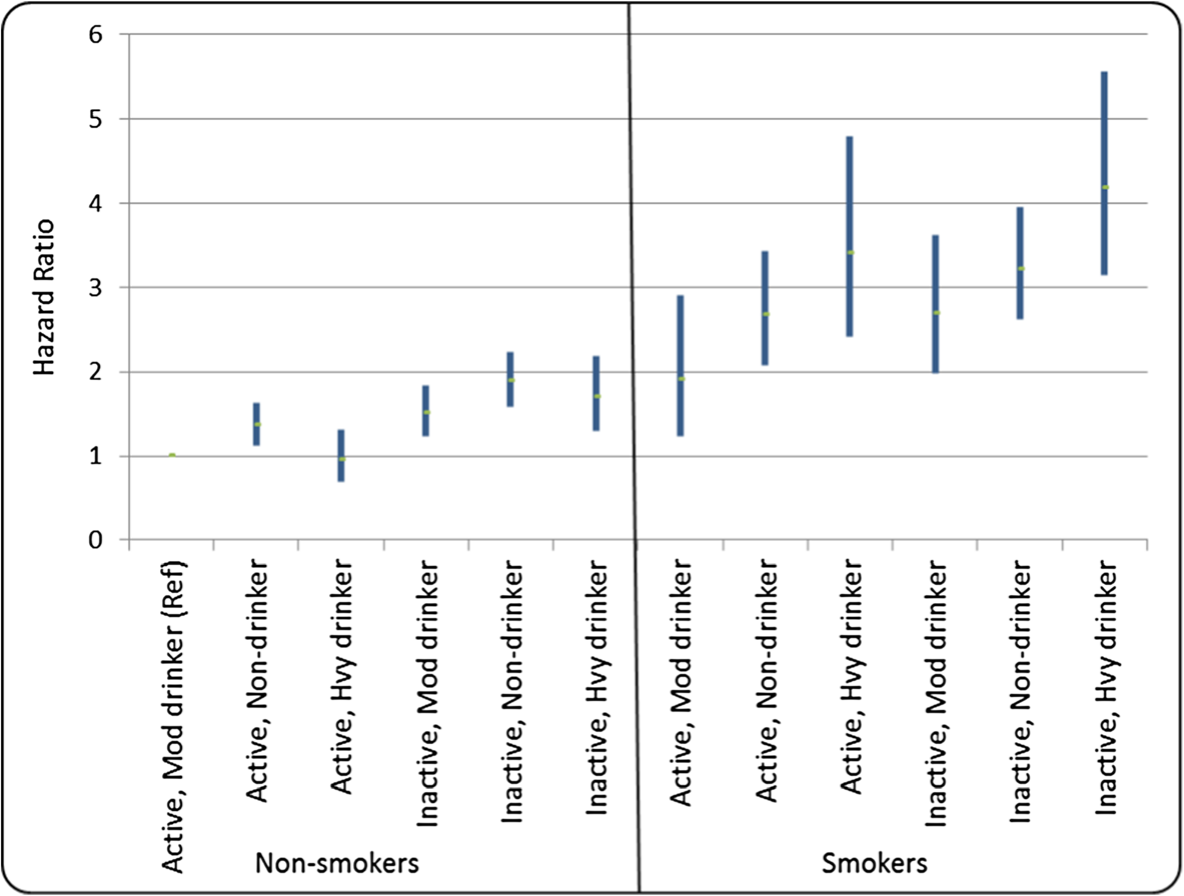
**Hazard ratios (and 99% CIs) depicting mortality risk associated with health behavior profiles measured in 1998** .

A similar degree of elevated mortality risk was observed among smokers who were also physically active and moderate drinkers (HR = 1.9, 99% CI = 1.2-2.9). Considerably higher risks were observed among smokers who were inactive and/or did not drink alcohol in moderation. More specifically, compared to the reference group, the risk of mortality was almost 3 times higher among smokers who were physically active non-drinkers (HR = 2.7, 99% CI = 2.1-3.4) and among smokers who were inactive moderate drinkers (HR = 2.7, 99% CI = 2.0-3.6). Mortality risk was more than 3 times higher among smokers who were inactive non-drinkers (HR = 3.2, 99% CI = 2.6-3.9), as well as smokers who were physically active heavy drinkers (HR = 3.4, 99% CI = 2.4-4.8). Mortality risk was elevated by more than 4-fold among smokers who were also inactive heavy drinkers (HR = 4.2, 99% CI = 3.1-5.6). Figure 1 provides a visual depiction of the mortality risk associated with each behavioral profile, and highlights the heterogeneity of estimates across this collection of profiles.

The results from Table 2 also indicate that the mortality risk of each behavioral profile was somewhat reduced among the older age group compared to the middle-aged group; however, a similar pattern of elevated risk within each profile was found in both age groups. Furthermore, additional analyses (not shown here) reveal that the mortality risk associated with the profile characterized by smoking, heavy drinking, and physical activity was stronger for men than women (*p* < .05); however, no other gender differences were found.

Table 3 presents results from several models using different reference groups in order to examine how the mortality risk of each behavior varies within different behavioral profiles. The first column of Table 3 shows that within a profile characterized by physical activity and moderate drinking, smoking compared to non-smoking was associated with a near doubling of mortality risk. Moreover, this column of Table 3 indicates that smoking was associated with an elevated risk of mortality, regardless of the other behaviors of one’s profile. Still, the risks of smoking varied as a function of other behaviors. For example, smoking had particularly strong effects among heavy drinkers. For those who were physically active and heavy drinkers, smoking was associated with 3.6 times the mortality risk compared to non-smokers (HR = 3.6, 99% CI = 2.4-5.4), and the risk of smoking was also relatively high among physically inactive heavy drinkers (HR = 2.5, 99% CI = 1.8-3.4).

Results in the second column of Table 3 focus on the mortality risks associated with physically inactive profiles. These results suggest that the risk associated with inactivity was more evident among non-smokers than smokers. Among non-smoking moderate drinkers as well as non-smoking non-drinkers, inactivity was associated with an increase of 40-50% in the risk of dying; among non-smoking heavy drinkers, inactivity elevated mortality risk by 80%. Among smokers, physical inactivity was associated with a slight elevation of risk (20%) only for those who were also non-drinkers.

The final two columns of Table 3 present the mortality risks associated with alcohol use within the context of different behavioral profiles. Among non-smokers, mortality risk was 30-40% higher among non-drinkers compared to moderate drinkers for both those who were physically active, and those who were inactive. Among smokers who were physically active, non-drinking was associated with a 40% increase in mortality risk.

The models assessing the association between heavy drinking and mortality also show variation across different behavioral profiles. Within the context of an otherwise healthy behavioral profile, heavy drinking was not associated with an elevated risk for mortality. In addition, heavy drinking was not associated with elevated mortality risk within a non-smoking, inactive, profile. But, within the context of behavioral profiles characterized by smoking, heavy drinking was associated with increases in mortality risk of between 60% and 80% (HR = 1.6, 99% CI = 1.1-2.2 for physically inactive smokers, and HR = 1.8, 99% CI = 1.1-2.9 for physically active smokers).

### Supplemental analyses

These results suggest that the mortality risk of smoking is strongest among heavy drinkers, and the risk of heavy drinking is strongest among smokers. Is the act of smoking simply more damaging to health when combined with heavy drinking, or do smokers who also drink heavily exhibit different patterns of smoking behavior compared to smokers who are moderate or non-drinkers? Likewise, do heavy drinkers who also smoke exhibit different drinking patterns compared to those who do not smoke? To help address these questions, Table 4 presents the average number of cigarettes smoked per day and the average number of years smoked, for each smoking profile. Also the average number of binge drinking episodes during the past 3 months is presented for each heavy drinking profile. Data come from the 1998 HRS survey.

**Table 4:** Comparisons of smoking and drinking patterns across profiles: smoking plus heavy drinking versus others

	**Cigarettes per day**		**Years smoked**		**Binge drinking, prior 3 months**	
	**Mean (SD)**		**Mean (SD)**		**Mean (SD)**	
Health Behavior Profiles		^a^		^a^		^a^
**Smoker, active, heavy drinker**	**14.1 (10.1)**	**---**	**38.5 (7.3)**	**---**	**16.3 (27.2)**	**---**
Smoker, active, moderate drinker	10.7 (9.1)	***	36.6 (9.8)			
Smoker, active, non-drinker	12.5 (9.9)		37.8 (10.4)			
Non-smoker, active, heavy drinker					8.9 (19.7)	***
		^b^		^b^		^b^
**Smoker, inactive, heavy drinker**	**16.3 (12.7)**	**---**	**40.6 (9.2)**	**---**	**16.7 (28.4)**	**---**
Smoker, inactive, moderate drinker	11.7 (9.1)	***	39.6 (7.7)			
Smoker, inactive, non-drinker	11.7 (8.7)	***	41.0 (10.2)			
Non-smoker, inactive, heavy drinker					9.3 (20.7)	***

*P ≤ .05; ** *P* ≤ .01; *** *P* ≤ .001.

^a^ Mean difference compared to the smoker, active, heavy drinker profile.

^b^ Mean difference compared to the smoker, inactive, heavy drinker profile.

The mean differences presented in Table 4 suggest that smokers who were also heavy drinkers tended to smoke more cigarettes per day than smokers who drank moderately or not at all. At the same time, however, smokers who drank heavily did not have significantly longer histories of smoking. With regard to binge drinking, results indicate that heavy drinkers who also smoked participated in binge drinking sessions at close to twice the rate of non-smoking heavy drinkers (e.g., mean of 16.3 for the smoker, active, heavy drinker profile vs. 8.9 for the non-smoker, active, heavy drinker profile, p < .001).

## Discussion

By examining the later life mortality risk associated with various combinations of health behaviors, this study revealed several important findings that have not previously emerged from studies that have assessed health behaviors individually or as undifferentiated components of a general health behavior index. For example, our findings indicate that the mortality risk associated with smoking varies according to the other health behaviors of an individual. Indeed, when paired with moderate drinking and a physically active lifestyle, the mortality risk of smoking was substantial, but also relatively modest compared to the risk associated with other smoking profiles. This trend was apparent both for adults aged 51–65 and aged 66+ at baseline, with some indications of an attenuation of risk in the older age group.

Using data from adults aged 20 and older in the NHANES, Ford et al. [11] found a similar pattern of relatively modest risk among smokers with healthy diets and adequate physical activity compared to smokers with unhealthy diets and inadequate physical activity. Our findings suggest that this trend continues into older ages, even when the overall magnitude of risk of smoking is likely to have declined due to selective survival. That is, while health behavior profiles involving smoking continue to pose similar patterns of risk on survival during the later stages of the life course, it appears likely that the selective survival of the most robust individuals in later life results in a slight mitigation of the level of risk that is imposed by profiles that include smoking.

The profile characterized by smoking in combination with heavy drinking exhibited the highest mortality risk of all of the health behavior profiles, both for the full sample and each age group. In particular, whereas smoking appears to double mortality risk on average, the risk of smoking compared to non-smoking reaches 2.5-fold within an inactive, heavy drinking profile, and as much as 3.6-fold within a physically active, heavy drinking profile. This finding of a compounding impact between smoking and heavy drinking is consistent with recent findings based on a sample of middle-aged Scottish men [46]. In that study, approximately 25% of the men who smoked and drank heavily did not survive to the age of 65. The current findings suggest that, even after selecting out those who died before reaching later life, the combination of smoking and heavy drinking continues to be the riskiest profile at advanced ages.

The joint effect of smoking and heavy drinking may represent the consequences of an interaction of risk that occurs when individuals combine these two particular behaviors [37]. However, according to our supplemental analyses, the smoking patterns of individuals who also drank heavily were actually different from smokers who drank less frequently. In particular, the findings from these analyses indicate that current levels of smoking, but not the duration of smoking, were higher among those who also drank heavily. Moreover, these findings suggest that among heavy drinkers, episodes of binge drinking were much more frequent among those who also smoked as compared to non-smokers. Thus, it appears as if the combination of smoking and heavy drinking in late life may be particularly risky, not just because of the accumulation, or interaction, of risk associated with these behaviors, but also because later life adults who combine these behaviors exhibit a more fundamental pattern of behavior, or lifestyle, which is vulnerable to ill health. Similar findings suggesting that smoking is particularly harmful when it takes place within the context of other lifestyle risks (e.g., stress) or social disadvantages (e.g., racial minority status) have recently been reported [38].

At the same time, however, when considering smoking and drinking together with physical activity, our findings suggest that inactivity poses a mortality risk for later life adults, but primarily within the profiles containing other healthy habits, such as non-smoking. This finding is inconsistent with recent findings showing that among adults aged 17 years and older from the NHANES III Mortality Study, physical inactivity in combination with an otherwise healthy lifestyle carries negligible risk [10]. Our findings, however, are consistent with findings from the Physicians’ Health Study [36], and as such, may be an indication of the particularly important role that physical activity plays for middle-aged and older adults. Still, our findings also suggest that the risk posed by physical inactivity is diminished, or perhaps just overshadowed, among later life adults with otherwise high risk profiles.

This type of overshadowing may also be evident when comparing the effects of alcohol abstinence across the various profiles. In both middle-aged and older adults, profiles involving moderate drinking were associated with lower mortality risks compared to both non-drinking and heavy drinking profiles. Additionally, the average independent mortality risks of both alcohol abstinence and heavy drinking were elevated compared to moderate drinking, a finding that is consistent with prior studies [49]. However, the current findings also show that the benefits of moderate drinking relative to abstinence seem to be present primarily within the context of an otherwise healthy behavioral profile [50], while the benefits of moderate relative to heavy drinking become evident only within profiles that include smoking.

This latter finding suggests that while heavy drinking, on average, is associated with elevated mortality risk, this behavior poses minimal risk to mortality when combined with other healthy behaviors (i.e., non-smoking), but substantial risk when combined with other unhealthy behaviors (i.e., smoking). Recent data from over 5,700 Scottish men aged 35–64 similarly shows that the mortality risk of heavy drinking is primarily evident only among current smokers [46]. Our findings again suggest that this same pattern of risk is likely to extend throughout the population of older adults.

### Study limitations

A primary limitation of this study involves the measurement of health behaviors. For example, the measure of smoking used in creating the 12 behavioral profiles did not distinguish between levels of smoking (e.g., heavy vs. light) or smoking history, both of which may have important implications for mortality risk [46,51]. Distinguishing levels of smoking, or different smoking histories, when creating the behavioral profiles would have resulted in a substantially greater number of distinct profiles with prohibitively small sample sizes associated with some of them. For this reason, data on smoking levels and histories were only utilized in post hoc analyses. Also, our measure of physical activity was based on a single survey item regarding vigorous physical activity, with just two response options. Thus, moderate activity, which is also associated with survival benefits [52], could not be captured. Furthermore, because dietary behavior was not measured in the HRS, this important aspect of health lifestyles could not be included in the current study [13]. Finally, an additional study limitation is that we used health behavior measures from baseline only, and thus did not attempt to account for changes in behavior that may have taken place during the follow-up period. Such behavior changes are difficult to interpret in mortality studies because they could be either a cause or a consequence of changing mortality risk. Still, future research focusing on associations between changes in health behavior profiles and mortality risk in the aging population is needed.

## Conclusions

Despite these limitations, this study lends important insight into the impact of health behaviors on survival during later life. Previous research has indicated the value of examining the joint effects of multiple behaviors, in particular, by showing the added value, in terms of survival, that individuals can achieve by maximizing the number of healthy behaviors in which they engage, and minimizing the number of risky behaviors. The current study extends this line of research by focusing on the mortality risk of specific combinations of health behaviors, and by focusing on older adults. On the basis of this approach, it appears as if behavioral profiles that combine multiple forms of substance use and misuse in later life, such as smoking and heavy drinking, are associated with particularly high levels of mortality risk, in both middle-aged and older adults. On the other hand, the mortality risk associated with heavy drinking within non-smoking profiles was minimal, while the risk of physical inactivity was primarily evident among non-smokers.

From a public health standpoint, these findings are important because they provide an indication of the importance that key health behaviors continue to have among those who have survived past age 50, while also providing information about which specific combinations of risk behaviors are most threatening at this stage of life. Within the context of a rapidly aging population, this type of information should be valuable for setting intervention priorities to promote healthy aging. What remains to be seen, however, is if these same behavior combinations that are responsible for extending or reducing late life survival, are equally influential with respect to expanding or compressing late life morbidity.

## Competing interests

The authors declare that they have no competing interests.

## Author’ contributions

BAS and NA conceptualized the study, interpreted the results, and wrote the manuscript. BAS analyzed the data. Both authors read and approved the final manuscript.

## Pre-publication history

The pre-publication history for this paper can be accessed here:

http://www.biomedcentral.com/1471-2458/12/803/prepub
